# DGH-GO: dissecting the genetic heterogeneity of complex diseases using gene ontology

**DOI:** 10.1186/s12859-023-05290-4

**Published:** 2023-04-26

**Authors:** Muhammad Asif, Hugo F. M. C. Martiniano, Andre Lamurias, Samina Kausar, Francisco M. Couto

**Affiliations:** 1 Biomedical Data Science Lab, Department of Bioinformatics and Biotechnology, Government College University Faisalabad, Faisalabad, 38000 Pakistan; 2 LASIGE, Departamento de Informática, Faculdade de Ciências, Universidade de Lisboa, Lisboa, Portugal; 3 Instituto Nacional de Saúde Doutor Ricardo Jorge, Avenida Padre Cruz, 1649-016 Lisbon, Portugal; 4 Department of Computer Science, Aalborg University, Ålborg, Denmark; 5 DeepOmicsVision, Avenue de Luminy, 13009 Marseille, France; 6 BioISI – Instituto de Biosistemas e Ciências Integrativas, Faculdade de Ciências, Universidade de Lisboa, 1749-016 Lisboa, Portugal; 7 NOVA LINCS, NOVA School of Science and Technology, Lisboa, Portugal

**Keywords:** Unsupervised learning, Dimension reduction, Gene ontology, Functionally similarities, Semantic similarity, Genetic heterogeneity, Neurodevelopmental disorders

## Abstract

**Background:**

Complex diseases such as neurodevelopmental disorders (NDDs) exhibit multiple etiologies. The multi-etiological nature of complex-diseases emerges from distinct but functionally similar group of genes. Different diseases sharing genes of such groups show related clinical outcomes that further restrict our understanding of disease mechanisms, thus, limiting the applications of personalized medicine approaches to complex genetic disorders.

**Results:**

Here, we present an interactive and user-friendly application, called DGH-GO. DGH-GO allows biologists to dissect the genetic heterogeneity of complex diseases by stratifying the putative disease-causing genes into clusters that may contribute to distinct disease outcome development. It can also be used to study the shared etiology of complex-diseases. DGH-GO creates a semantic similarity matrix for the input genes by using Gene Ontology (GO). The resultant matrix can be visualized in 2D plots using different dimension reduction methods (T-SNE, Principal component analysis, umap and Principal coordinate analysis). In the next step, clusters of functionally similar genes are identified from genes functional similarities assessed through GO. This is achieved by employing four different clustering methods (K-means, Hierarchical, Fuzzy and PAM). The user may change the clustering parameters and explore their effect on stratification immediately. DGH-GO was applied to genes disrupted by rare genetic variants in Autism Spectrum Disorder (ASD) patients. The analysis confirmed the multi-etiological nature of ASD by identifying four clusters of genes that were enriched for distinct biological mechanisms and clinical outcome. In the second case study, the analysis of genes shared by different NDDs showed that genes causing multiple disorders tend to aggregate in similar clusters, indicating a possible shared etiology.

**Conclusion:**

DGH-GO is a user-friendly application that allows biologists to study the multi-etiological nature of complex diseases by dissecting their genetic heterogeneity. In summary, functional similarities, dimension reduction and clustering methods, coupled with interactive visualization and control over analysis allows biologists to explore and analyze their datasets without requiring expert knowledge on these methods. The source code of proposed application is available at https://github.com/Muh-Asif/DGH-GO

**Supplementary Information:**

The online version contains supplementary material available at 10.1186/s12859-023-05290-4.

## Background

Complex diseases manifest with a broad range of phenotype, mediated by hundreds of genetic variants that differ in their structure, mode of inheritance, and frequency of occurrence. Complex diseases such as Autism Spectrum Disorder (ASD) present a heterogeneous phenotype and genotype that hinders the establishment of phenotypic and genotypic associations, thus, restricting the applications of modern precision medicine and biomedicine approaches.

A large number of genetic variants, including Single Nucleotide Variants (SNVs) and Copy Number Variants (CNVs) contribute to genetic heterogeneity of several disease by altering different functionally important genes [1, 2]. For example, CNVs are associated with several complex diseases such as ASD, Schizophrenia, Epilepsy, and Intellectual Disability (ID) [3–8]. Previous studies have reported that CNVs contribute to phenotypic heterogeneity of complex diseases [9].

Genetic variant(s) disrupting a gene that follows a certain path across different biological levels may cause a spectrum of disease phenotypes. One example of such gene is syndromic genes. Multiple genes located at different genomic locations disrupted by different variants converge at certain biological level to generate a specific disease phenotype. The group of genes converging on certain biological level tends to have similar biological functional. In case of complex disease each group of genes may consequent into a distinct biological mechanisms and clinical outcome.

Due to recent advances in genomic technologies, it is possible to accurately detect genetic variants in a larger population. Consequently, consortium based studies have genotyped thousands of patients and have revealed a large amount of genetic variants [6–8, 10, 11], providing opportunities to infer their biological mechanism, functional similarities and disease relevance. Enhanced understanding of functional interactions of putative disease causing genes forming a distinct group could pave a way to the establishment of phenotypic and genotypic associations. However, it is challenging to unravel the functional relationships of these potential disease candidates. Additionally, variants like CNVs span several genes, thus further intensifying the problem of putative causal gene identification.

Pipelines have been developed to annotate and infer biological functions of genetic variants using existing resources such as Gene Ontology (GO) [12]. Asif et al. presented a systematic pipeline that include both pre and post processing steps to infer biological mechanisms for rare CNVs [12]. Also, there exist the state-of-the-art methods to predict ASD putative casual genes [13, 14].

However, the phenotypic manifestations of predicted disease genes are not reproducible on other datasets. Furthermore, to better understand the disease prognosis and to facilitate the precision medicine currently, one of the fundamental challenges is to identify groups of functionally similar genes that govern specific and distinct disease traits.

Complex diseases exhibit multiple etiologies, indicating the role of hundreds of genetic variants. These genetic variants hardly act in isolation and studies have shown that putative disease causing genetic variants converge on common biological processes, indicating a functional relationship. It has also been hypothesized that functionally related genes tend to develop similar phenotypes. Studies have used Protein–Protein Interaction (PPI) networks and genes co-expression network to identify modules of genes, which may or may not lead to similar phenotype development [15].

One alternative to networks is ontological resources such as GO that contains more specific and higher level biological details for genes. GO is an ontological resource with three type of vocabularies namely biological process, molecular functional and cellular locations. The GO resource is an extensive and uniform biological resource with frequent updates. Functional similarities between genes can be assessed by applying semantic similarity measures on GO terms associated for the targeted genes. Previously, Asif et al. has highlighted the importance of genes functional similarities, computed using GO and semantic similarity measures [14]. The proposed classifier outperformed existing tools in predicting disease genes [14].

**Fig. 1:**
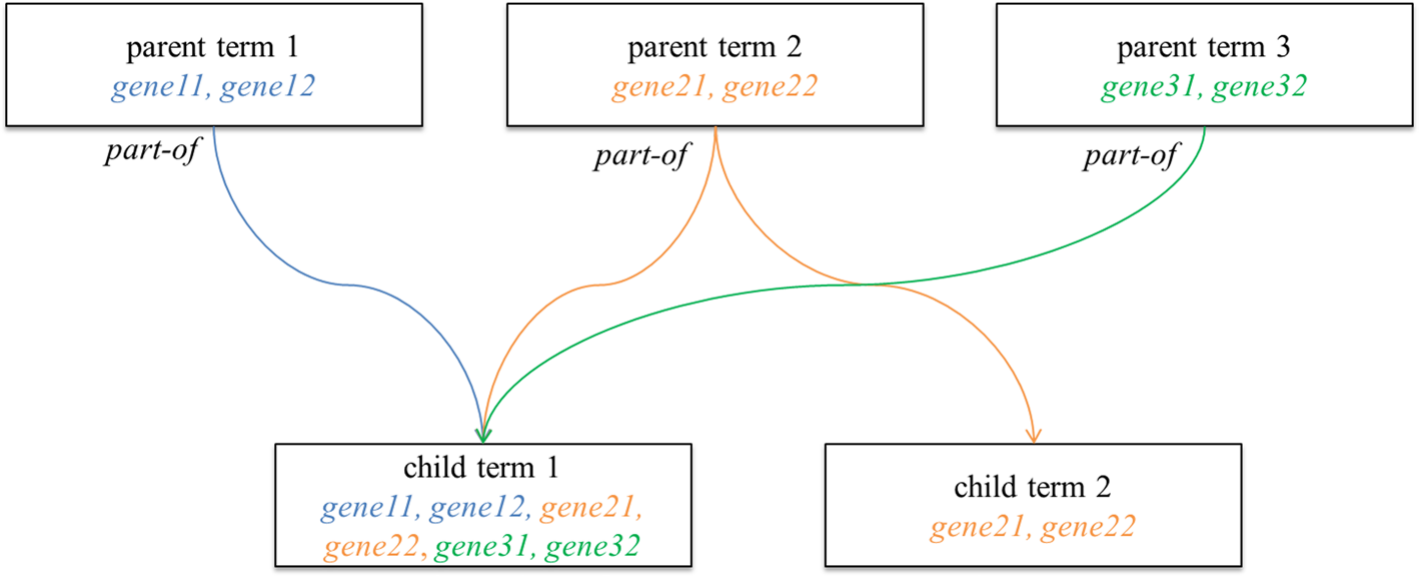
Graphical representation of GO DAGs structure. Child term 1 has three ancestor terms (parent term 1, 2, and 3) with a *part-of* relationship. In such relationship, all the genes annotated with child term 1 are coming from its ancestor’s terms

The GO is the most widely used and extensive ontological resource in biology and consists of three controlled vocabularies i.e. biological process, molecular function and cellular location. GO implements a Directed Acyclic Graph (DAG) structure where nodes, presenting GO terms, are linked through parent–child relationships. In such relationships, child nodes inherit all the annotations (genes in this study) associated with all of its parent nodes through a specific relationship. An example of such a relationship is “part-of '' meaning the child node is a subset of the parent node (Fig. 1).

Semantic similarity measures use this structure to assess the similarities between nodes i.e. GO terms (Fig. 1). Resnik [16], Wang [17], Lin [18] and Jiang [19] are frequently used semantic similarity measures. Semantic similarity measures use the GO structure to find the functional similarities between genes and score it in the range of 0–1, where 0 indicates that genes are highly distant and 1 means genes are identical. The score closer to 1 indicates functional similarity between genes.

In this study we proposed a pipeline, called Dissecting the Genetic Heterogeneity using GO (DGH-GO) with a graphical user interface. DGH-GO hypothesized that putative disease causing genes tend to converge on similar biological processes and pathways, indicating the functional relationship between them. Also, functionally similar genes may lead to similar or identical phenotype(s). The DGH-GO allows biologists to analyze a list of genes emerged from their large scale genomic studies or created from known disease databases to study the shared biological mechanisms among different diseases or conditions.

The proposed study contributes as follows:

1. In depth analysis of genes. DGH-GO allows the users to perform extensive analysis i.e. discovering the biological convergence patterns or functional associations for input genes. The analysis starts from descriptive statistics, calculation of semantic similarities, and dimension reduction, followed by clustering of genes.
2. Higher level of biological functional details: DGH-GO applies semantic similarity measures on GO to compute gene’s functional similarities that allow inferring the biological convergence patterns of putative or known disease causing genes.
3. User friendly: DGH-GO is the first interactive web application that aims for the identification of clusters comprising functionally similar genes. Furthermore, it allows users to apply different dimension reduction methods to visualize the input data in a reduced dimension. Currently, DGH-GO supports the analysis by five semantic similarity measures (coupled with four aggregate functions), four different dimension reduction and clustering methods. The users are free to choose any method to analyze their input genes. The ease of applying multiple methods in DGH-GO also makes it easier for users lacking computing skills to compare the performance of different methods for their targeted genes.
4. Biological applications:
  - The proposed methodology was applied on genes disrupted by rare CNVs in ASD patients. The analysis through DGH-GO revealed that rare CNVs disrupting the genes in ASD patients converge on biological processes and form multiple clusters that were enriched for distinct but ASD related pathways and phenotype(s), confirming the multi-etiological nature of ASD.
  - In addition, DGH-GO was also tested to dissect the shared etiology of complex diseases. The analysis showed that genes involved in multiple disorders tend to aggregate in separate clusters.

## Implementation

**Fig. 2:**
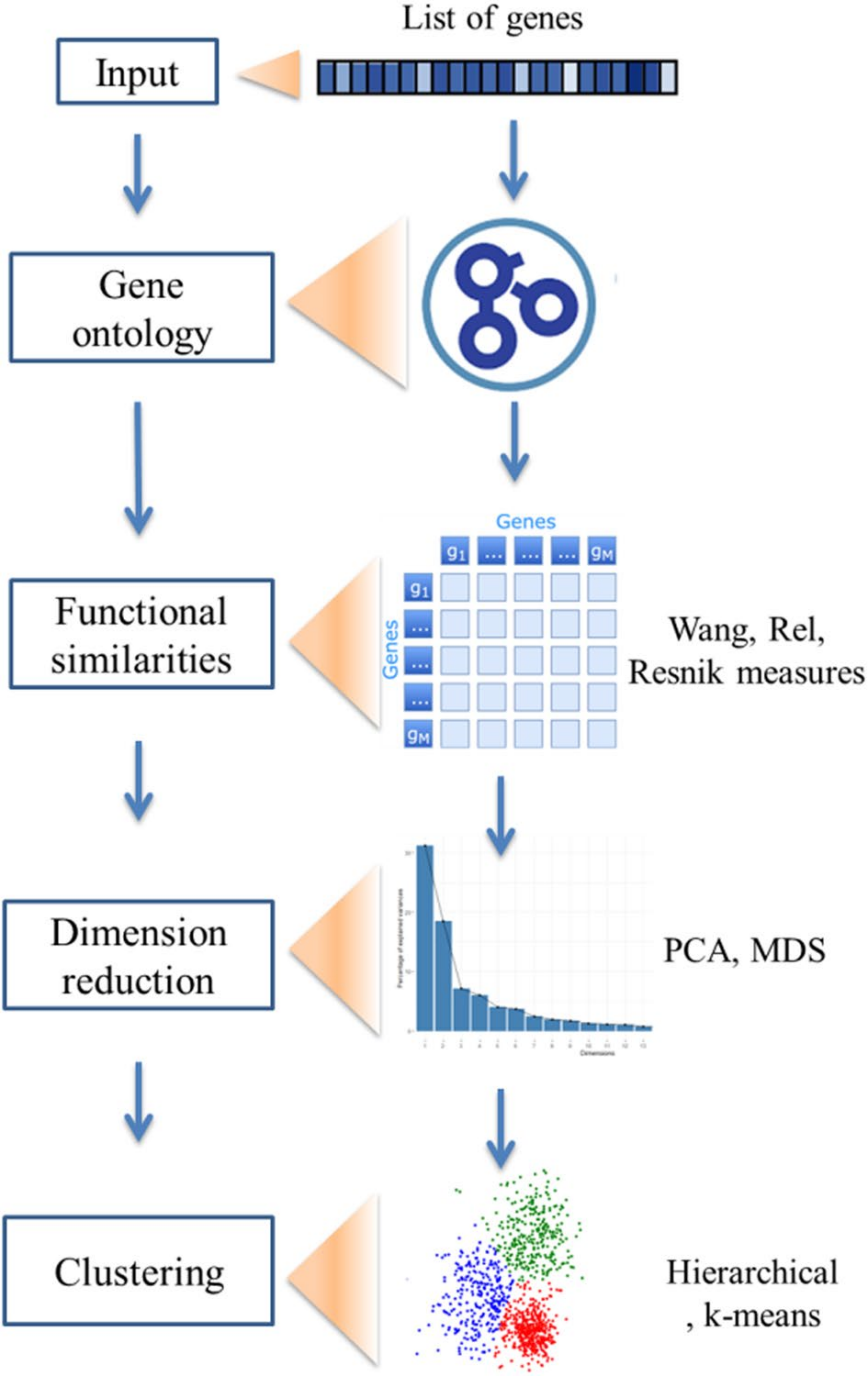
Graphical representation of workflow of DGH-GO. *MDS* multidimensional scaling

DGH-GO uses gene’s functional similarities to infer the convergence pattern of putative/known disease causing gene. Figure 2 provides a graphical overview of DGH-GO workflow. The DGH-GO consists of three modules. The DGH-GO is developed in R Shiny. Shiny is an R package, which has been recently widely used to develop interactive web applications for bioinformatics data analysis. For example, Shiny applications have been developed for single-cell RNA seq and bulk RNA seq [20, 21].

The 1.7.1 shiny version was installed in R of version: 4.2.0. To calculate the functional similarities of genes the GOSemSim R package [22, 23] was used. DGH-GO uses the GOSemSim R package to apply semantic similarity measures and output the functional similarity matrix.

### Dataset

For the case study of disease signature identification, genes disrupted by rare CNVs in ASD patients were obtained from Sander et al. [22]. Sander et al. reported rare CNVs disrupting the 3698 genes in 3802 patients diagnosed with ASD. For the second case study to explore the shared etiology of complex diseases, a list of genes known for ID, DD and EE was obtained from Zhang et al. [23]. Genes causing ASD were downloaded from the SFARI gene database (https://gene.sfari.org/). SFARI gene database is a well curated source of ASD genes which is periodically updated by domain experts. The expert researchers curate genes associated with ASD based on available references in literature. The SFARI gene database is a public database and ASD genes can be downloaded freely as a CSV file.

## Results

### Overview of DGH-GO

DGH-GO aims to dissect the genetic heterogeneity by identifying the clusters of functionally similar genes that may develop a distinct biological mechanism and disease phenotypes.

Identification of clusters containing the functionally similar genes is based on functional similarities, computed by applying semantic similarity measures on GO information for the targeted genes. Figure 2 shows the graphical representation of DGH-GO workflow.

DGH-GO consists of three modules, (1) Functional Similarities (FunSim) module; (2) Dimension Reduction (DimReduct) module; and (3) Clustering the Functional Similarities (ClusFunSim) module.

1. Functional Similarities (FunSim) module

The FunSim module calculates pairwise functional similarities among input genes. The functional similarities of genes can be determined by using controlled biological vocabularies, such as GO. Input genes may be a list of genes disrupted by genetic variants such as CNVs or SNPs. Alternatively, it can also be a list of genes involved in different complex diseases. For example, ASD and ID are complex NDDs. Genes causing ASD are also involved in ID. *MBD5* is a syndromic gene and associated with both ASD and ID [24]. To estimate the functional similarities of genes, semantic similarity measures namely Resnik, Wang, Lin, Rel, and Jiang has been widely used. Most often, these measures are based on annotation details of the common ancestor terms. Some measures, in particular proposed by Wang utilizes the DAG structure of GO to compute the functional similarities of genes. Each measure has its own reported limitations, strengths and applications. The measures that utilize the annotation statistics of common ancestor, for example Resnik, Jiang, Rel and Lin are based on information content (IC). The IC of a GO term is defined by the negative logarithm of its probability of occurrence in a given collection of GO terms (Eq. 1).

1 $$IC\left(GO term\right)=-\mathrm{log}\left(p\left(GO term\right)\right)$$

As showed in Fig. 1, a GO term may have more than one ancestor terms. Due to the possibility of multiple parents for each term, it is possible that two terms can have shared parents by multiple paths in the DAG of GO. The measures based on IC compute the functional similarity of two terms by estimating the information content of their closest common ancestor terms, referred as most informative common ancestor (MICA). The Resnik similarity measure computes relatedness between two GO terms by utilizing the information content of the MICA. Resnik measure to calculate similarity between two GO terms is defined as:

2 $${Sim}_{Resnik}\left({Go term}_{1}, {Go term}_{2}\right)= IC\left(MICA\right)$$

Another measure proposed by Lin is also based on IC and for GO terms it is defined as:

3 $${Sim}_{Lin}\left({Go term}_{1}, {Go term}_{2}\right)= \frac{2 X IC(MICA)}{IC\left({Go term}_{1}\right) +IC({Go term}_{2})}$$

The Rel measure combines Lin and Resnik measures to determine the relatedness between two GO terms. The mathematical expression for Rel measure is provide in Eq. 4.

4 $${Sim}_{Rel}\left({Go term}_{1}, {Go term}_{2}\right)= \frac{2 X IC(MICA)(1-p\left(MICA\right))}{IC\left({Go term}_{1}\right) +IC({Go term}_{2})}$$

Jiang similarity measure also computes similarity between GO terms by comparing their IC values. Equation 5 provides the mathematical formula for Jiang measure.

5 $${Sim}_{Jiang}\left({Go term}_{1}, {Go term}_{2}\right)= 1-\mathrm{min}(1, IC\left({Go term}_{1}\right) + IC\left({Go term}_{2}\right)-2 X IC(MICA))$$

The Wang similarity measure does not use IC values of GO terms, instead it utilizes the DAG of GO. Wang measure first computes the semantic value (SV) for a GO term by estimating the contributions of its neighboring GO terms. A set of neighboring terms is created using the DAG structure.

6 $${Sim}_{wang}\left({Go term}_{1}, {Go term}_{2}\right)= \frac{ \sum_{t \epsilon {T}_{Go term1 }\cap {T}_{Go term2` }} {S}_{{Go term}_{1}}\left(t\right)+{S}_{{Go term}_{2}}\left(t\right) }{SV\left({Go term}_{1}\right)+SV({Go term}_{2})}$$

SV is calculated as:

7 $$SV\left({Go term}_{1}\right)= \sum_{t \epsilon {T}_{Go term1 }} {S}_{{Go term}_{1}}\left(t\right)$$

where $t$ refers to terms having child terms in a DAG structure.

The description and mathematical derivation of similarity measures is provided in the context of GO and was adapted from GoSemSim vignette, available through Bioconductor.

The FunSim module employs these similarity measures (Resnik, Wang, Lin, Rel, and Jiang) to compute genes functional similarities. Semantic similarity measures utilize the structure of the ontology resource to calculate similarities for biomedical or non-biomedical entities. The most commonly used ontologies in life sciences are GO, Human Phenotype Ontology (HPO), and Disease Ontology (DO).

To calculate the similarities at gene level, semantic similarities scores of GO terms (associated with input genes) are aggregated into a final score, ranging between 0 and 1 through aggregate functions. The available aggregate functions are max, avg, rcmax and BMA.

The max combining function calculates the functional similarity among all GO terms and chooses the maximum score from created scoring matrix. The avg function computes the average functional similarity for all GO terms. The rcmax calculates the column and row scores separately and chooses the maximum value from created set of values. The BMA combining function is based on best match average approach, meaning final score is calculated by the average of all maximum similarities across each row and column.

DGH-GO allows users to choose one semantic similarity measure from Resink, Rel, Wang, Lin, and Jiang and one aggregate function. The selected measure along with aggregated function is used to calculate functional similarities for input genes list.

**Fig. 3:**
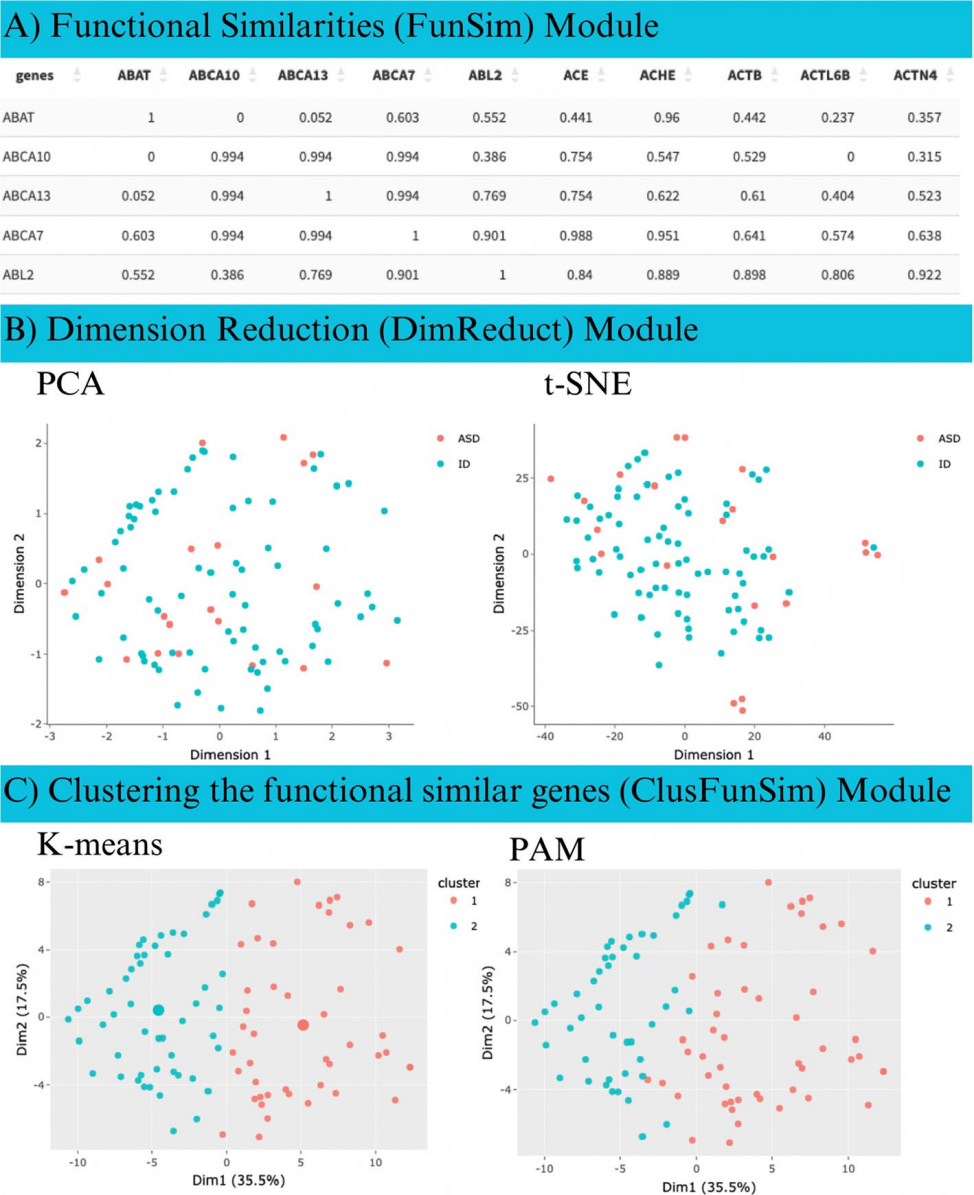
Output of DGH-GO. **A** Semantic similarity matrix showing the functional similarities of input genes; **B** dimension reduction of input genes; **C** clustering of genes using functional similarities

The output of the FunSim module is a squared semantic similarity matrix, coupled with genes and their known or putative diseases (Fig. 3A). The resulting similarity matrix is interactive and permits searching, sorting and export operations.

(2) Dimension Reduction and Visualization (DimReduct) module

Large scale genomic studies of complex diseases such as ASD produce a large list of putative disease causing genes, disrupted by genetic variants in patients. Also, studying the shared etiology of several complex diseases such as ASD and ID can generate a large functional similarities matrix. The DimReduct module provides the option of applying different dimension reduction methods. Currently, DGH-GO supports Principal Component Analysis (PCA), Principal Coordinate Analysis (PcoA) and t-SNE methods to reduce dimensions of functional similarity matrix. The input to the DimReduct module is the functional similarities matrix generated by the FunSim module. The users can decide the number of dimensions and may also choose the specific dimensions for visualization of data in 2D space. The projections of functionality matrix with user defined number of dimensions are downloadable for future use. The interactive 2D visualization of dimensionally reduced data is also provided (Fig. 3B).

(3) Clustering the functional similar genes (ClusFunSim) module

The ClusFunSim module applies different unsupervised clustering methods either on functional similarities matrix coming from FunSim module or dimensionally reduced data, output of DimReduct module. The users are free to define inputs for clustering methods. The ClusFunSim module contains four different clustering methods, namely K-means, PAM, hierarchical and Fuzzy. These clustering methods have been widely used in bioinformatic data analysis approaches [17]. Along with deciding the input data type, the users can also set the number of clusters for each clustering method.

DGH-GO allows users to validate the clustering results. The Silhouette measure is used to find the optimum number of clusters for the input functional similarity matrix of genes. Silhouette value estimates the cohesion and separation of identified clusters. It measures how much one instance is similar to its cluster and is separated from another cluster. Mathematically, the Silhouette value for a gene $\left(gene A\right)$ is defined as:

8 $$Silhouette(gene A)= \frac{b\left(gene A\right)-a(gene A)}{\mathrm{max}(a\left(gene A\right), b(gene A)}$$

$Silhouette(gene A)$ is the Silhouette value for gene A and $a(gene A)$ is the average distance of $gene A$ from all the genes in same cluster. Whereas, $b\left(gene A\right)$ is the average distance between the $gene A$ and other genes present in the nearest cluster. The Silhouette value ranges from − 1 to 1, where − 1 indicates highly dissimilar and 1 means highly similar. An instance is considered as wrongly clustered if it has Silhouette score of 0 or less than 0.

Silhouette value for a whole cluster can also be estimated from Silhouette values of its members, genes. For this purpose, for each gene in a cluster, its average distances from its cluster member genes are computed ($a\left(gene A\right)$ using Eq. 8). Similarly, for each gene in a cluster, $b\left(gene A\right)$ is calculated. Lastly, a Silhouette value for a cluster is calculated by averaging the Silhouette values of each gene present in cluster (Eq. 9).

9 $$Silhouette(cluster)= \frac{\sum Silhouette (gene A)}{\mathrm{N}}$$

N is the number of genes. To provide an overview of the Silhouette values for each input instance (genes in this study), DGH-GO provides a Silhouette plot for all the clusters. The Silhouette plot also shows the wrongly clustered genes (Silhouette value < 0) for each cluster. Furthermore, it also provides the mean of Silhouette values across all clusters by drawing a red line in Silhouette plot. Additionally, a downloadable and quantitative table containing inter and intra clusters validation is also shown to user. The table includes diameter and size of cluster, average distance between and inside of clusters, separation and Silhouette score for each cluster.

The ClusFunSim module outputs the 2D clustering plot, plot for optimum number of clusters, a qualitative table showing the intrinsic and extrinsic validation of clusters, and a downloadable tabular representation of genes and their assigned clusters (Fig. 3C). The interactive plots and tables are available for each clustering method, hence providing the possibility of comparing different clustering methods for a given dataset.

## Applications

The biological applicability of DGH-GO was assessed by two case studies:

1. Dissecting the multi-etiological nature of ASD i.e. inferring the biological convergence of putative ASD causing genes disrupted by rare CNVs
2. Understanding the shared etiology of complex diseases
3. Dissecting the multi-etiological nature of ASD

ASD is a complex neurodevelopmental disorder and difficult to diagnose due to phenotypic heterogeneity. ASD is defined by repetitive behavior and social deficits [25]. It is hypothesized that ASD candidate genes converge on biological processes, creating distinct groups of functionally similar genes. Each group follows a different or shared biological mechanism to generate a specific phenotype. To test this hypothesis, DGH-GO was applied to genes spanned by CNVs in ASD patients, which were obtained from Sanders et al. [26]. Rare CNVs disrupting 3698 genes in 3802 individuals diagnosed with ASD were collected from Sanders et al. [26]. The semantic scores were available for 2457 genes. Rel semantic similarity measure with the Max aggregation function was used to compute the functional similarities among genes. PCA was applied to functional similarity matrix to visualize it in a lower space. Additional file 1 contains supplementary details of PCA and clustering results. First nine PC components that explained the majority of the variance were selected for clustering (Additional file 1: Fig. S1).

Silhouette measure was used to find the optimum number of clusters, which were 16 clusters (Additional file 1: Fig. S2). A tabular representation of clustering results validation can be found in Additional file 1: Table S1. Silhouette analysis of each individual cluster showed the presence of outliers (Additional file 1: Fig. S3). A gene occurring in one cluster with a silhouette score less than 0 was considered as outlier. All the outliers (N = 376) were excluded and optimum number of clusters was re-computed. The new refined data gave 14 optimum numbers of clusters, again using the silhouette score as a criterion (Additional file 1: Fig. S4).

The first 6 components of PCA explained majority of the variance in data (Additional file 1: Fig. S5). The variance explained by each component was estimated. The elbow plot was used to visualize the variance explained by the first 50 components (Additional file 1: Fig. S5). 1^st^ PC component explained majority of the variance in data, which was declining for next components. The elbow plot shows that from the 7th PC component the change in explained variance is minimal. The difference in variance explained by 6th and 7th PC components was higher than the difference in variance explained by 7th and 8th components of PCA. Therefore, the first six PC components were selected for further analysis. The validation of all clusters is provided in Table 1 and Additional file 1 (Figs. S6 and S7). The validation of all clusters is provided in Table 1 and Additional file 1 (Figs. S6 and S7).

**Table 1:** The resultant clusters with their average Silhouette values

Cluster	Genes (N)	Cluster average Silhouette	Average Silhouette
1	192	0.187	0.327
2	355	**0.331**	
3	198	**0.279**	
4	179	**0.391**	
5	100	0.159	
6	280	**0.524**	
7	98	0.172	
8	84	**0.268**	
9	140	0.04	
10	34	**0.921**	
11	89	0.088	
12	142	**0.576**	
13	135	**0.261**	
14	55	**0.703**	

Silhouette value measures the cohesiveness and separation of a cluster and ranges from − 1 to 1. The clusters with bold average Silhouette values are stable and compact

Silhouette analysis of all the 14 clusters showed that 9 clusters (2, 3, 4, 6, 8, 10, 12, 13, and 14) are well separated and cohesive (Table 1). To annotate these compact and cohesive clusters of genes, functional enrichment analysis was performed separately for each cluster using Enrichr [27]. From Enrichr, KEGG database [28–30], DisGeNET [31], and mammalian phenotype ontology [32] was chosen for functional enrichment analysis. Additional files (2-6) contains the functional enrichment analysis results for each cluster. 

**Fig. 4:**
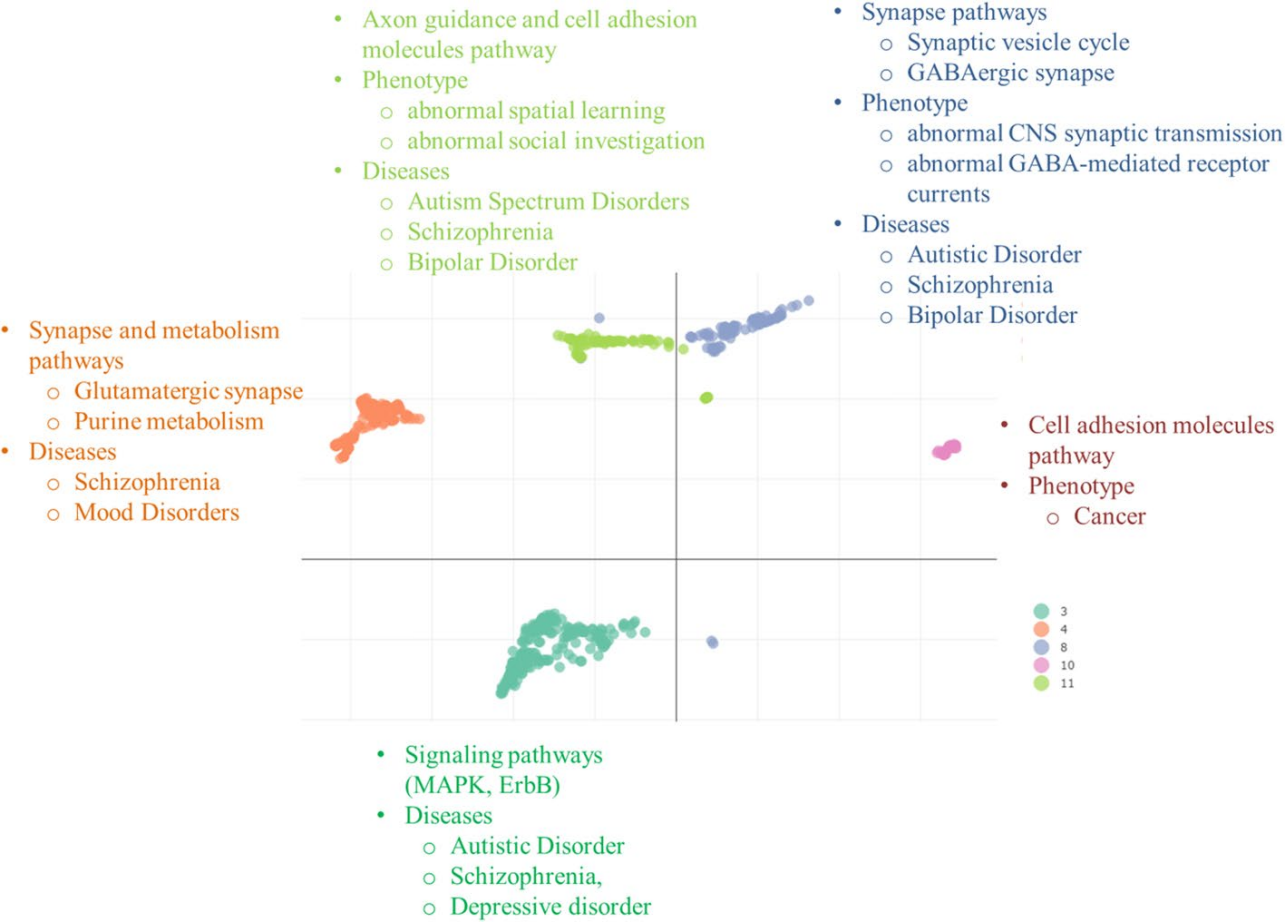
Clusters enriched for ASD related pathways or phenotypic terms. Cluster 3 genes N = 198, Cluster 4 genes N = 179, Cluster 8 genes N = 84, Cluster 10 genes N = 34, Cluster 11 genes N = 89

5 clusters (3, 4, 8, 10, and 11) out of 9 stable clusters were enriched for ASD related biological processes and pathways. They were also significantly associated with ASD phenotype and its co-occurring conditions such as mood disorders. The clusters enriched for ASD related terms were compact and consistent as indicated by Sil-widths values from Silhouette validation measure (Table 1). Other clusters were either unstable or not related to ASD (Table 1). Figure 4 shows the umap of 5 stable and ASD related clusters and also enriched pathways and phenotypic terms for each cluster.

Cluster 11, containing 89 genes, was enriched for Axon guidance and cell adhesion molecules related pathways. The 7 and 5 genes from cluster 11 were significantly associated with *abnormal spatial leaning* and *abnormal social investigation phenotype* respectively. This cluster was also linked with Autism Spectrum Disorder, Schizophrenia and Bipolar Disorder from DisGeNET database [31, 33]. Top and ASD related enriched pathways, phenotypic terms and disease terms for cluster 11 are shown in Fig. 4 (Additional file 2).

84 genes gathered in cluster 8 were specifically enriched for synapse related pathways. The most significant pathway was *Neuroactive ligand-receptor interaction*. 8 and 6 genes were associated with *Synaptic vesicles cycle* and *GABAergic synapse* pathways respectively. Furthermore, genes of cluster 8 were also associated with *abnormal CNS synaptic transmission* and *abnormal GABA-mediated receptor current* in mouse. Functional enrichment analysis against the DisGeNET database showed that *Autistic disorder*, *Schizophrenia*, and *Bipolar Disorder* were enriched in cluster 8 (Fig. 4 and Additional file 3).

Cluster 4, containing 179 genes, is a mixed cluster and was involved in synapse and metabolism related pathways. The most statistically significant pathway for cluster 4 was *Neuroactive ligand-receptor interaction* pathway. Other ASD related pathways such as *Glutamatergic synapse* and *purine metabolism* was also associated with this cluster. *GABAergic synapse* pathway was marginally significant for cluster 4. However, no phenotypic term was significantly enriched for this cluster. From DisGeNET, *Schizophrenia* and *Mood Disorder* were significantly enriched for cluster 4 (Fig. 4 and Additional file 4).

Cluster 3 was a signaling cluster and exhibited the *MAPK and ErbB signaling pathways* but no statistically significant phenotype term was found for the genes of this cluster. From DisGeNET resource, the genes of clusters 3 were associated with *Autistic Disorder*, *Schizophrenia*, and *Depressive Disorders* (Fig. 4 and Additional file 5).

Cluster 10, with few genes (N** = **34) was enriched for *Cell Adhesion molecules*. However, no phenotype term was found for this cluster. Similarly, no ASD related terms were found from DisGeNET. Mainly this cluster was enriched for Cancer related terms from DisGeNET resource (Fig. 4 and Additional file 6).

### 2) Understanding the shared etiology of complex diseases

To show another application of DGH-GO in dissecting the shared etiology of complex disorders, genes known for ASD, Developmental Disorders (DD), Intellectual Disability (ID), and Epileptic Encephalopathy (EE) were obtained from Zhang et al. [34]. The aim of this case study was to test the multi-etiological nature of ASD, instead of discussing the extent of shared etiology of different NDDs. Functional similarity matrix was of 427 (ASD = 74, ASD + DD = 10, ASD + DD + EE = 1, ASD + DD + ID = 19, ASD + DD + ID + EE = 2, ASD + ID = 2, DD = 204, DD + EE = 4, DD + ID = 25, DD + ID + EE = 3, ID = 63, EE = 19, ID + EE = 1).

PCA was applied to functional similarity matrix of complex diseases and the first six PC components were chosen for clustering as they explained the majority of the variance (Additional file 1: Fig. S5). Silhouette score indicated the existence of 22 optimum numbers of clusters (Additional file 1: Fig. S6). Hierarchical clustering on PC components was performed with 22 clusters using the Ward linkage criterion. The 14 clusters were consistent, stable and compact (Table 2) and were used for further analysis.

**Table 2:** The resultant clusters of genes, known for multiple disorders with their average Silhouette values

Cluster	Genes (N)	Cluster average Silhouette	Average Silhouette
1	9	**0.298**	0.273
2	11	**0.309**	
3	32	0.18	
4	18	0.15	
5	29	0.082	
6	21	**0.359**	
7	14	**0.278**	
8	22	**0.458**	
9	26	**0.328**	
10	16	0.179	
11	9	0.147	
12	40	**0.333**	
13	21	**0.274**	
14	8	**0.576**	
15	18	0.115	
16	14	0.135	
17	14	0.186	
18	25	**0.277**	
19	22	**0.311**	
20	23	**0.386**	
21	23	**0.383**	
22	12	**0.306**	

Silhouette value measures the cohesiveness and separation of a cluster and ranges from − 1 to 1. The clusters with bold average Silhouette values are stable and compact

Each selected cluster contains outliers (genes clustered with a silhouette score of < 0) (Additional file 1: Fig. S7). In contrary to previous analysis of dissecting the multi-etiological nature of ASD, no exclusion criterion was applied to outliers. The reason is the authenticity of genes as they are known candidate genes for complex diseases. All the genes were known for their associations with diseases. In the analysis of rare CNVs disrupting genes, outliers were expected because not all rare CNVs span disease causing genes.

**Fig. 5:**
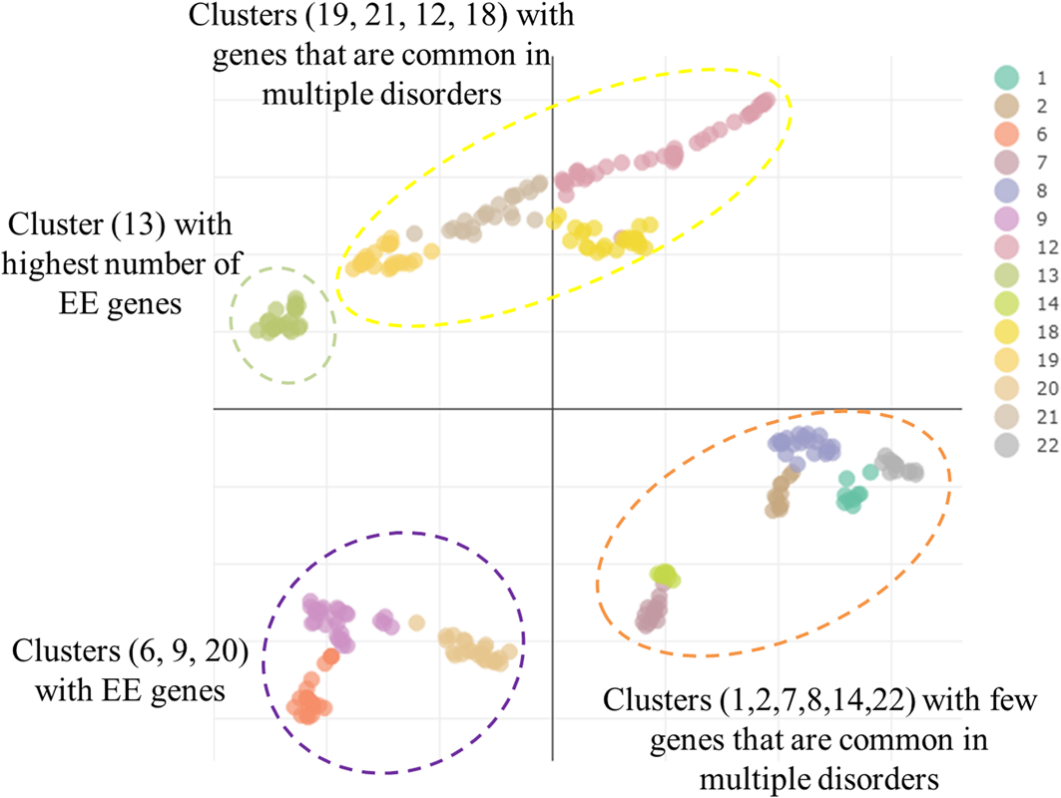
Umap of clusters obtained from genes involved in multiple disorder

14 stable clusters are shown in Fig. 5 and Table 2. The genes shared by multiple disorders aggregated on the same clusters and are closer to each other in 2D space. The clusters with the largest number of shared genes of ASD, DD, ID, and EE were 1, 2, 7, 8, 14, and 22 (Fig. 5). Clusters number 6, 9, and 20 contain higher proportions of EE genes than other clusters. Cluster 13 contains a larger number of EE genes than all of the other clusters. Clusters (19, 21, 12, and 18) contain genes which are not shared by multiple disorders. For example, cluster 1 contains genes known for ASD and ID but there is no evidence that these genes are common in both diseases.

## Discussion

Due to advances in genomic technologies collection of large number of variants, disrupting several protein coding genes in a larger population is possible. One of the most recent challenges in post-genomic era is the identification of biological convergence patterns of hundreds of variants that are involved in complex diseases.

The highly precise biological convergence patterns or groups of functionally related genes converge to specific biological mechanisms that may be responsible for distinct disease phenotype(s). Several methods have been proposed for disease putative causal gene prediction or identification [12–15, 35].

However, efforts to understand how hundreds of genes interact to develop disease specific phenotype or a spectrum of phenotype have been lagging behind. For example, there are unanswered questions such as do all the hundreds of candidate genes of complex diseases interact with each other to develop disease? How all the candidate genes or a subset of genes with similar function converges on targeted functional pathways to develop a specific disease outcome? Does the multi-etiological nature of complex diseases emerge from distinct or related convergence pattern of disease causing genes?

Furthermore, complex diseases also share etiology, meaning that at certain point of disease phenotype development the different diseases follow similar biological mechanisms. What are those shared mechanisms and do genes involved in different diseases are functionally closer to each other or distant from other genes that are disease specific? These are burning and challenging questions for understanding the etiology of complex genetic disorders.

In this study, we have proposed an approach, named as DGH-GO with a graphical interface to find the clusters of functionally similar genes that may exhibit distinct biological mechanisms that could generate a specific disease phenotype(s). Previously network approaches using PPI and gene expression profiles have been used to detect sub-communities in a network [15].

Previously network approaches using PPI and gene expression profiles have been used to detect sub-communities in a network [15]. Linkage methods of network approaches assume that the close neighbours of disease causing gene products possess higher likeliness of being involved in similar disease phenotype [36, 37]. Oti, M. et al. predict the putative disease causing genes by utilizing the protein–protein interactome [37]. Over the time, several structured and well maintained resources for PPI data such as STRING [38] and BioGRID [39] have emerged. However, PPI resources differ on criteria and objectives for data collection, analysis and manipulation. For example, STRING is using a numeric score to rank the protein–protein interactions whereas BioGRID is a binary score-based database. Therefore, the selection of the PPI resource could affect the final conclusion [40].

Network approaches have also been applied on gene expression networks. Langfelder et al. developed an R package, called weighted correlation network analysis (WGCNA) [41]. WGCNA determines the clusters of genes that are correlating at the expression level. Such methods aiming for the identification of correlation networks have effective applications in discovering the putative disease-causing genes. Network approaches employed on either PPI or gene expression profiles are focused on identification of hub entities (genes or proteins) or sub modules in a larger network which may contribute to disease development. Barabási et al. has reviewed the network approaches for studying human diseases [42].

Alongside with the information provided by PPI and gene expression, GO is a resource which is specially designed for enrichment analyses and is rich in biological details. Deciding the data resource to propose a disease gene prediction method is difficult. Each data set has its own strengths and weaknesses, which is beyond the scope of this study. The recently emerged data integration methods can be used to integrate these datasets to leverage their strengths. However, integration of diverse datasets is challenging. GO is uniform resource that has DAGs structure and each new entry follows the same structure, which makes it uniform, easy to use, share, and interpret. DGH-GO takes advantage of GO uniformity and extensiveness, coupled with detailed biological description and uses it to find functional similarities among genes. These functional similarities between genes are fed to unsupervised learning methods such as dimension reduction and clustering methods to discovery the converging patterns of putative disease causing genes.

Another strong aspect of DGH-GO is the freedom of choosing the methods for the analysis. Several methods have been routinely used for genetic data analysis and most of them require programming experience, thus limiting the role of domain experts (biologists or clinical doctors) in analyzing their generated data. Also, choosing a method from an extensive list of available methods can be a daunting task for people lacking bioinformatics or data analytical skills.

DGH-GO is an easy to use interactive application that allows users to apply multiple methods and compare their performance. For example, a user can perform different dimension reduction methods and perform different clustering methods either on semantic similarity scores or one of the dimensionally reduced data. DGH-GO is an open source application, which allows people with programming skills to modify it according to their need.

The proposed application was evaluated on two different datasets of complex diseases. First rare CNVs spanning putative ASD-causing genes were analyzed to explore the genetic heterogeneity of ASD. The DGH-GO analysis showed that putative ASD-causing gene groups into different clusters and each cluster displayed a different biological mechanism. Multiple clusters of varying functionality were expected because ASD occurs with multiple independent etiologies and it has been hypothesized that independent phenotype(s) emerge from different biological mechanisms. Separate phenotypic analysis of each cluster also showed that clusters differing on biological mechanisms also differ on phenotype. ASD is a neurodevelopmental disorder and mainly affect nervous system, more specifically synapse transmission. The genes of cluster 8 identified by DGH-GO were significantly enriched for synapse related term indicating the genes of this are involved in ASD core symptoms. Moreover, this cluster was also enriched *abnormal CNS synaptic transmission* and *abnormal GABA-mediated receptor phenotype* terms further confirming that it is highly associated with ASD. Similarly other clusters such as cluster 3 were enriched for signalling pathways. Several lines of evidence have reported that ASD is linked with Signalling pathways such as ERb and MapK, further confirming the role of cluster 3 genes in ASD. The analysis of DisGeNET also indicated that genes of cluster 3 are associated with ASD. Cluster 3 and 4 are also enriched for Axon guidance and Synapse. Previous studies have the associations of these pathways with ASD. The enriched terms for the 5 clusters are related to ASD, which is in line with previously published literature [6, 7, 12–14, 43, 44].

To show the biological significance of DGH-GO, it was also applied to genes known for multiple disorders such as ASD, ID, EE, and MD. The analysis of genes known for multiple disorders clustered in to same groups, this was inline with previous studies showing that genes involved in multiple disorders are aggregated in the same clusters [34].

It was also observed that EE clustered distantly from other clusters, for example, cluster 13, containing the highest number of EE gene, organizes itself distant from others. It was expected and consistent with existing knowledge of EE because it is not a truly neurodevelopmental disorder like ASD and ID.

The proposed application, DGH-GO, is limited to the GO data resource. Recently, we proposed a knowledge graph (KG) to explore the disease gene associations [45]. KG integrated data from GO and DisGeNET, a publicly accessible database of diseases and genes. The created KB consists of 93,657 unique entities (28,243 genes; 21,623 diseases; 11,170 molecular functions; 4183 cellular components; and 28,438 biological processes) and 59 relationship types (900,442 gene-disease associations; 715,550 gene-GO annotations; and 89,593 GO ontology relationships). The proposed KG showed the importance of data integration and fusion in personalized medicine. Other researchers have reviewed data integration and data fusion methods and also heighted their importance in both genomic medicine and precision medicine [46, 47]. However, effective translation of these big and complex methods to the biology domain is still challenging.

## Conclusion

In this study we have proposed a pipeline to dissect the genetic heterogeneity of complex diseases by identifying the groups of similar genes that may lead to distinct phenotype(s). For this we have applied semantic similarity measures, dimension reduction and clustering methods. A graphical user interface, named as DGH-GO is also available for users lacking programming experience. DGH-GO enables biologists control over analysis and allows them to infer biological convergence patterns or functional associations for a targeted gene set. Additionally, the possibility of applying different methods in an interactive way, coupled with higher level of biological information helps biologists to enhance their mechanistic understanding for an underlying problem.

A case study of ASD-associated genes targeting putative disease causing variants confirmed the multi-etiological nature of ASD and showed that identifying clusters of functionally similar genes could hint about the existence of distinct biological convergence patterns leading to specific biological mechanisms and phenotypes. The results from the analysis of ASD genes were consistent with literature. In another case study, DGH-GO confirmed the hypothesis of shared etiology in NDDs. The analysis of genes involved in multiple disorders exhibited common convergence patterns, indicating an overlapping genetic etiology.

Currently, DGH-GO is limited to GO. However, to complement the limitations of GO, the future version of it will be focused on integrating other biological data resources such as PPI, gene expression profiles, disease ontology and human phenotype ontology.

### Availability and requirements

Project name: DGH-GO

Project home page: https://github.com/Muh-Asif/DGH-GO

Operating system(s): Platform independent

Programming language: R and Shiny

Other requirements: None

License: MIT License

Any restrictions to use by non-academics: None

System specification: 8 GB of RAM and core i7 CPU.

## Supplementary Information

**Additional file 1:** The results of PCA and clustering analysis applied to the functional similarity matrix.

**Additional file 2:** Enrichment analysis of genes from cluster 11. KEGG pathways database, mammalian phenotype ontology and DisGeNET database were used to find the enriched pathways, phenotype ontology and disease terms, respectively.

**Additional file 3:** Enrichment analysis of genes from cluster 8. KEGG pathways database, mammalian phenotype ontology and DisGeNET database were used to find the enriched pathways, phenotype ontology and disease terms, respectively.

**Additional file 4:** Enrichment analysis of genes from cluster 4. KEGG pathways database, mammalian phenotype ontology and DisGeNET database were used to find the enriched pathways, phenotype ontology and disease terms, respectively.

**Additional file 5:** Enrichment analysis of genes from cluster 3. KEGG pathways database, mammalian phenotype ontology and DisGeNET database were used to find the enriched pathways, phenotype ontology and disease terms, respectively.

**Additional file 6:** Enrichment analysis of genes from cluster 10. KEGG pathways database, mammalian phenotype ontology and DisGeNET database were used to find the enriched pathways, phenotype ontology and disease terms, respectively.

## Data Availability

For the case study of disease signature identification, genes disrupted by rare CNVs in ASD patients were obtained from Sander et al. [21]. Sander et al. reported rare CNVs disrupting the 3698 genes in 3802 ASD patients. For the study of shared etiology of complex diseases, a list of genes known for, ID, DD and EE was obtained from Zhang et al. [23]. ASD genes were obtained from SFARI gene databases (https://gene.sfari.org/). DGH-GO can be downloaded from https://github.com/Muh-Asif/DGH-GO.

## Abbreviations

NDDs: Neurodevelopmental disorders
GO: Gene Ontology
ASD: Autism Spectrum Disorder
SNVs: Single Nucleotide Variants
CNVs: Copy Number Variants
ID: Intellectual Disability
PPI: Protein-Protein Interaction
DAG: Directed Acyclic Graph
DGH-GO: Dissecting the Genetic Heterogeneity using GO
FunSim: Functional Similarities
DimReduct: Dimension Reduction
ClusFunSim: Clustering the Functional Similarities
DO: Disease Ontology
HPO: Human Phenotype Ontology
PCA: Principal Component Analysis
PcoA: Principal Coordinate Analysis
DD: Developmental Disorders
ID: Intellectual Disability
EE: Epileptic Encephalopathy
